# Epidemiology of hip fracture and the development of a FRAX model for Uzbekistan

**DOI:** 10.1007/s11657-020-00792-7

**Published:** 2020-07-29

**Authors:** O Lesnyak, S Ismailov, M Shakirova, N Alikhanova, A Zakroyeva, L Abboskhujaeva, H Johansson, NC Harvey, E McCloskey, JA Kanis

**Affiliations:** 1 Mechnikov North West State Medical University, St. Petersburg, Russia; 2 Republican Medical Center for Endocrinology, Tashkent, Uzbekistan; 3 Ural State Medical University, 3 Repina street, Yekaterinburg, Russia; 4 Mary McKillop Institute for Health Research, Australian Catholic University, Melbourne, Australia; 5 MRC Lifecourse Epidemiology Unit, University of Southampton, Southampton, UK; 6 Centre for Metabolic Bone Diseases, University of Sheffield, Sheffield, UK

**Keywords:** FRAX, Fracture probability, Osteoporosis epidemiology, Hip fracture, Uzbekistan

## Abstract

**Summary:**

A prospective population-based survey in a region of the Republic of Uzbekistan determined the incidence of fractures at the hip. The hip fracture rates were used to create a FRAX® model to facilitate fracture risk assessment in Uzbekistan.

**Objective:**

This paper describes the epidemiology of hip fracture in the Republic of Uzbekistan that was used to develop a country-specific FRAX® tool for fracture prediction.

**Methods:**

During a 1-year (2016/17) prospective population-based survey in the Pap district of the Republic of Uzbekistan, hip fractures were prospectively identified from hospital registers, trauma centres and primary care and community sources. Age- and sex-specific incidence of hip fracture and national mortality rates were incorporated into a FRAX model for Uzbekistan. Fracture probabilities were compared with those from neighbouring Kazakhstan and Kyrgystan.

**Results:**

Approximately 41% of hip fracture cases did not come to medical attention, and two thirds of patients overall were not admitted to hospital. The incidence of hip fracture applied nationally suggested that the estimated number of hip fractures nationwide in persons over the age of 50 years for 2015 was 16,764 and is predicted to increase more than three-fold to 60,272 in 2050. FRAX-based probabilities were higher in Uzbekistan than Kazakhstan or Kyrgystan.

**Conclusion:**

The FRAX model should enhance accuracy of determining fracture probability among the Uzbek population and help guide decisions about treatment.

## Introduction

Osteoporosis is a common, chronic and costly condition; its principal clinical consequence is fracture. In Europe, the annual cost of fractures associated with osteoporosis exceeded € 37 billion in 2010 [1]; disability due to osteoporosis was greater than that caused by any single cancer, with the exception of lung cancer, and was comparable or greater than that lost to a variety of chronic noncommunicable diseases, such as rheumatoid arthritis, asthma and high blood pressure-related heart disease [2, 3]. Fortunately, a wide variety of treatments is available that favourably affect bone mass and thereby decrease the risk of fractures associated with osteoporosis [4]. The use of such interventions by health care practitioners is assisted by instruments that assess patients’ fracture risk to optimize clinical decisions about prevention and treatment. The most widely used web-based tool FRAX® (https://www.sheffield.ac.uk/FRAX/) meets these requirements and computes the 10-year probability of fragility fractures based on several common clinical risk factors and, optionally a DXA scan result [5, 6]. FRAX models are available for 66 countries in 2020 covering more than 80% of the world population at risk [7] and have been incorporated into more than 100 guidelines worldwide [8].

The availability of FRAX has stimulated studies that can be used for the generation of new FRAX models. Specific examples include Brazil, Mexico and Turkey [9]. The present study is a component part of the Multicenter Multinational population-based Study in Eurasian Countries (EVA study or ЭВА, in Russian). The broad aim of the study was to provide epidemiological information on fracture risk so that FRAX models could be created for Russia [10], Armenia [11], Belarus [12], Moldova [13], Kazakhstan [14], Kyrgyzstan [15] and Uzbekistan. The present report describes the epidemiology of fractures at the hip in Uzbekistan and the generation of a country-specific FRAX model.

## Methods

The republic of Uzbekistan is a landlocked country in turn bordered by five landlocked countries: Kazakhstan, Kyrgyzstan, Tajikistan, Afghanistan and Turkmenistan. Uzbekistan has an area of 447,400 km^2^ (172,700 mile^2^) with a population estimated at 33.5 million in 2020 [16]. Uzbeks comprise a majority (80%) of the total population. Other ethnic groups include Russians 2%, Tajiks 5%, Kazakhs 3%, Karakalpaks 2.5% and Tatars 1.5% (1996 estimates).

The Pap district of Uzbekistan was selected as the catchment area to document the incidence of hip fracture. The district was chosen for its geographic location: in the North and in South-west, the Pap district borders with the Republics of Kyrgyzstan and Tajikistan. In the North-West, the district borders with the Tashkent region, but it is only possible to get access through the mountain pass to Namangan by car for 8 h of the day. In the South, the Pap region borders the Ferghana region, but patients from neighbouring districts can get medical care there only for a payment. These characteristics were expected to optimize the delineation of a catchment area and minimize the probability that hip fracture patients would be treated outside the region.

The population at risk (that is the total population of the Pap region) proved difficult to determine in that three sources of data were identified that gave very disparate estimates. According to State Department of Statistics [17], the population comprised 193,267 residents in the year of study, of whom 55,098 (29%) were 40 years of age or older. By contrast, the Pap region Department of Health care estimated the population age 40 years or older at 103,481 [Nematjon Kirgizbayev (2015) Personal Communication to Said Ismailov, 09 September 2015]. Finally, an intermediate estimate of 69,384 was derived from information that individuals age 40 years or more in the Pap district comprised 0.8% of the total Uzbek population and that the age and sex distribution in Pap was very similar to that in the whole country [18]. We discarded the first two estimates since the apparent incidence of hip fracture derived there from was the lowest and highest worldwide, respectively.

Thus, the catchment population comprised 69,384 residents (32,784 men and 36,600 women) age 40 years or older representing 0.8% of the population of Uzbekistan. The ethnic admixture of the Pap region is similar to that of Uzbekistan. The national population demography by sex and 5-year intervals was obtained from United Nations [16].

The prospective study, undertaken for 1 year from April 2016 until March 2017, was preceded by a training period following a directive from the Uzbekistan Ministry of Health to all medical units of Pap District with the requirement to improve the detection of osteoporotic fractures. A meeting and short training course was organized thereafter for all medical staff of the Pap region who dealt with trauma patients. This included all 27 general practitioners, radiologists, coroners, emergency physicians, traumatologists, surgeons and internists from the district. The aim of the meeting was to improve the recording and documentation of cases of hip fracture in order to develop a national FRAX model. Case report forms were developed to record the patient’s age, sex, place of residence, date, character of injury and ICD-10 code (S72.0, S72.1, S72.2). In addition, the official medical records of hip fractures in men and women from the central city hospital registers, the outpatient trauma unit data, GP data and all emergency service data and coroner cases were reviewed. We also looked for the patients from Pap district who were operated in the central hospital of the neighbouring Namangan region (no eligible cases were identified).

We also engaged home visiting nurses who were instructed to notify the traumatologists about each suspected case that had not been referred for hospital care. An orthopaedic surgeon subsequently examined all such cases. The diagnosis was verified clinically (all patients) and where possible, by radiographic examination (89% of cases). Additionally, we contacted the seven folk healers (tabibs) in the region who were asked to redirect all suspected patients to the Central hospital trauma centre of Pap district. Finally, we contacted the elders of the administrative communities (Mahalla) each covering about 1350–1500 residents. They identified housebound individuals who were subsequently visited at home by the district nurse.

The reason for accessing multiple sources of information including that from primary care was to identify patients with hip fracture who were not admitted to hospital. This strategy was necessary since many patients in Eastern Europe are not hospitalized because facilities for surgical management are limited so that hospital admission is not feasible. In Belarus, for example, 29% hip fracture cases did not come to hospital attention [12]. High rates of non-admittance have been reported in Armenia (44%) [11], Pervouralsk in Russia (27%) [10], Georgia (75%) [19], Kazakhstan (29%) [14] and Kyrgyzstan (50%) [15]. These missing cases from hospital discharge data reinforce a view that data on hip fracture based solely from hospital records are unreliable in this region of the world.

To avoid double counting, further admissions for the same fracture site in the observation time were excluded. In some documents, the fracture ICD-10 code was not specified. In such cases, radiographs were retrieved and fractures, if verified, were included in the database. Permanent residence in the region was a criterion for inclusion. High energy fractures were excluded (falls from greater than from a standing height). We excluded pathological fractures attributable to cancer with metastases or to multiple myeloma.

The age- and sex-specific incidence in 2016/2017 was applied to the population in 2015 to estimate the number of hip fractures nationwide. Additionally, future projections were estimated up to 2050 assuming that the age- and sex-specific incidence remained stable. Population demography was taken from the United Nations using the medium variant for fertility [20].

The data on hip fracture were used to construct the FRAX model. For other major osteoporotic fractures (clinical spine, forearm and humeral fractures), it was assumed that the age- and sex-specific ratios of these fractures to hip fracture risk found in Sweden were comparable to those in Kazakhstan. This assumption has been used for many of the FRAX models with incomplete epidemiological information. Available information suggests that the age- and sex-stratified pattern of fracture is very similar in the Western world, Australia and Eastern Europe [13, 21–23].

The development and validation of FRAX have been extensively described [5, 6]. The risk factors used were based on a systematic set of meta-analyses of population-based cohorts worldwide and validated in independent cohorts with over 1 million patient-years of follow-up. The construct of the FRAX model for Uzbekistan retained the beta coefficients of the risk factors in the original FRAX model with the incidence rates of hip fracture and mortality rates for Uzbekistan. National mortality rates used data from the World Health Organization for 2014 [24]. Ten-year fracture probabilities were compared to those of the neighbouring countries where a FRAX model was available (Kazakhstan and Kyrgyzstan).

In order to compare hip fracture probabilities with those of other regions of the world, the remaining lifetime probability of hip fracture from the age of 50 years was calculated for men and women, as described previously [25]. In the present analysis, values for Uzbekistan were compared with those of China (Hong Kong), Bulgaria, Canada, Denmark, Finland, France, Greece, Hungary, Kazakhstan, Kyrgyzstan, Mexico, Moldova, Poland, Portugal, Romania, Russia, Spain, Sweden, Turkey, Ukraine, United Kingdom and the United States (Caucasian) [13, 14, 26, 27].

## Results

**Fig. 1:**
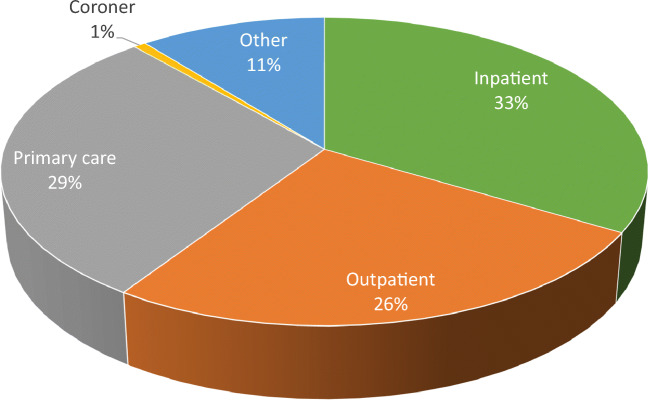
Proportion (%) of hip fracture cases admitted to hospital as an inpatient (inpatient), outpatient only at hospital (outpatient), treated only in primary care, identified through the coroner and identified by nurses, tabibs and community elders (other)

Over the period of 1 year, 140 low energy hip fractures were identified in men (*n* = 52) and women (*n* = 88) age 40 years or more. Of these cases, only 47 (33%) of hip fracture patients were hospitalized (Fig. 1). The hospitalized patients were treated with osteosynthesis or conservatively; only two patients underwent emergency hemiarthroplasty surgery.

Twenty-six percent of patients received only outpatient treatment by a traumatologist. The primary care physicians identified 29% of new hip fracture cases that were managed only in primary care. The home visiting nurses, tabibs and the Community Elders found 15 additional, otherwise unreported cases (11%).

The crude annual incidence of low-energy hip fracture in individuals age 40 years or more was 240/100,000 in women and 159/100,000 in men (female/male ratio = 1.6). Hip fracture incidence increased with the age up to the age of 90 years in both men and women (Table 1). The incidence in women rose more steeply with age than in men.

**Table 1:** Population at risk (2015), number of hip fractures (2016/2017) and annual hip fracture incidence (per 100,000) with 95% confidence intervals in the male (M) and female (F) population of the Pap district of Uzbekistan

Age (years)	Population		Number of hip fractures		M		F	
	M	F	M	F	Incidence	95%CI	Incidence	95%CI
40–44	7480	7760	2	1	27	3–97	13	0.3–72
45–49	6312	6768	3	4	48	10–139	59	16–151
50–54	5984	6632	2	4	33	4–121	60	16–155
55–59	4984	5536	5	8	100	32–234	145	62–285
60–64	3552	3968	6	7	169	62–368	176	71–364
65–69	1704	2064	8	13	470	203–925	630	335–1078
70–74	1104	1344	6	9	544	199–1183	670	306–1272
75–79	912	1208	7	12	768	309–1582	993	513–1736
80–84	480	752	7	13	1458	586–3005	1729	919–2958
85–89	216	400	5	11	2315	748–5406	2750	1371–4922
90+	56	168	1	6	1786	36–9964	3571	1310–7774
	32,784	36,600	52	88				

### Fracture projections

Assuming that the fracture rates in the Pap district were representative for the whole country, and based on the UN estimates of the Uzbek population for 2015 (31.3 million), the annual number of hip fractures in men and women age 50 years or older in Uzbekistan in 2015 was estimated at 16,764. The number of hip fractures is expected to increase progressively over calendar year with a greater than three-fold increase by 2050 (Table 2). A similar increase was noted for major osteoporotic fractures.

**Table 2:** Estimated total number of hip and major osteoporotic fractures (MOF) in men and in women age 50 years or older in 2015 projected up to 2050 in Uzbekistan

	2015	2020	2030	2040	2050
Hip fracture					
Men	5942	7131	10,277	15,063	20,761
Women	10,822	12,773	18,522	27,978	39,511
Men and women	16,764	19,904	28,799	43,041	60,272
MOF					
Men	19,262	22,846	32,182	45,245	59,383
Women	41,751	49,769	71,722	102,031	133,949
Men and women	61,013	72,615	103,904	147,276	193,332

**Fig. 2:**
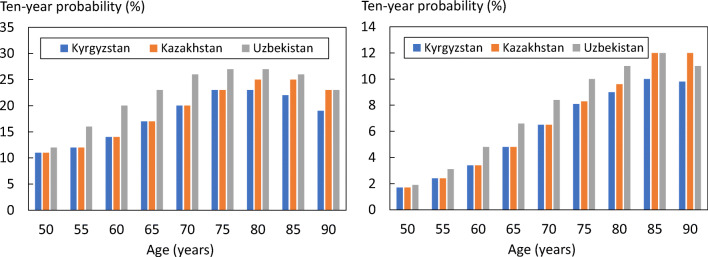
Ten-year probability of a major osteoporotic fracture (left hand panel) and hip fracture (right) in women with a prior fracture by age from Kyrgyzstan, Kazakhstan and Uzbekistan. Body mass index set to 25 kg/m^2^

The 10-year probability of major osteoporotic fracture and hip fracture in Uzbekistan, Kazakhstan and Kyrgyzstan is shown in Fig. 2 in women with a prior fracture by age. Ten-year probabilities in Uzbekistan were consistently, though moderately, higher than in Kazakhstan or Kyrgyzstan.

Lifetime probabilities for hip fracture are shown in Table 3.

**Table 3:** Lifetime probability of hip fracture in the Uzbek population from the age of 50 years compared with selected countries

Country	Lifetime risk at 50 years %	
	Women	Men
Sweden	25.6	11.0
Denmark	23.0	11.3
France	19.3	5.9
China (Hong Kong)	17.7	7.6
USA (Caucasian)	16.1	7.5
Turkey	15.9	3.6
Canada	15.5	5.8
Greece	15.4	6.8
Uzbekistan^a^	14.7	8.7
UK	14.4	5.0
Portugal	13.7	4.8
Finland	12.9	6.0
Kazakhstan	12.6	6.0
Spain	12.6	4.2
Kyrgyzstan	11.7	6.4
Bulgaria	11.2	4.4
Hungary	10.8	4.2
Mexico	10.6	5.0
Poland	10.1	4.2
Moldova	9.3	5.7
Russia	7.7	3.8
Romania	7.0	3.8
Ukraine	5.6	2.9

^a^Present study

## Discussion

This study documented the incidence of hip fracture in the Pap district of Uzbekistan. As expected, hip fractures were more frequent in women than in men. In both sexes, the incidence increased with age. Assuming that the regional incidence was similar to the national incidence, Uzbekistan belongs to the moderate-risk countries for hip fracture for men and women [26].

The number of hip fractures nationwide was estimated at 16,764 in 2015.

Demographic projections indicate that fracture burden is due to increase markedly in the future. It is estimated that the annual number of hip and other major osteoporotic fractures will increase more than three-fold over 35 years. The prediction is relatively robust in that all individuals who will be aged 50 years or more in 2050 are currently adults. However, these estimates may be conservative since they assume that the age- and sex-specific risk of hip fracture remains unchanged over this period. If the age- and sex-specific incidence of hip fracture increases, as has been registered in several countries [28], then the number of fractures may be more than doubled. Such projections are important for healthcare planning.

The access to all medical records in this study, including those from primary care, permitted the identification of patients with hip fracture who were not admitted to hospital. The reason for this strategy was the observation that many patients in Eastern Europe are not hospitalized because facilities for surgical management are limited so that hospital admission is not feasible [10–12, 14, 19]. The present study indicated that two thirds of hip fracture cases were not admitted to hospital. The treatment gap arises for many reasons including a lack of emergency orthopaedic surgeons. These findings are also important for healthcare planning and emphasize the importance of exploring care pathways in the design of epidemiological studies.

A minority of countries that have a FRAX model also have robust information on the risk of other major osteoporotic fractures. In the absence of such information, FRAX models are based on the assumption that the age- and sex-specific pattern of these fractures is similar to that observed in Malmo [20]. As already noted, this assumption has been shown to be safe in studies reported from many countries [13, 21–23, 29–31], despite differences in incidence between these countries [26]. This commonality of pattern is supported by register studies, which indicate that in those regions where hip fracture rates are high, so too is the risk of forearm fracture and spine fractures (requiring hospital admission) [32, 33].

The incidence of hip fracture was used to create a FRAX tool to compute the 10-year probabilities of hip and major osteoporotic fracture in Uzbekistan. Ten-year probabilities were marginally higher than in the neighbouring countries of Kazakhstan and Kyrgyzstan. Other neighbouring countries are Tajikistan, Afghanistan and Turkmenistan but no FRAX models are available to make comparisons.

The widespread availability of FRAX has resulted in its adoption in many practice guidelines worldwide [8]. The fracture probability equivalent to a woman with a prior fracture has been used as an intervention threshold in more than 30 countries. If the same threshold were applied to Uzbekistan, then intervention would be recommended with a probability of a major fracture that varied between 12 and 27% depending on age. The impact of such thresholds or alternative thresholds will require further study.

There are a number of additional limitations to this study. These include the relatively small sample size and, therefore, wide confidence intervals from which to compute fracture incidence and the difficulty in case verification. With regard to fracture incidence, we examined less than 1% of the Uzbek population from a single district. Thus, the extrapolation of this regional estimate to the entire country is an assumption that we were unable to test. In addition to large variations in fracture rates around the world, fracture rates may vary within countries. In addition to ethnic-specific differences [34], up to two-fold differences in hip fracture incidence have been reported using common methodology with the higher rates in urban communities including Croatia [35], Switzerland [36], Norway [37], Argentina [38] and Turkey [39]. A major limitation relates to uncertainties regarding the catchment population. Three estimates for the catchment population is unprecedented in our experience. The highest and lowest numerical estimates would have provided incidence estimates for hip fracture that were the lowest and highest, respectively, world-wide. Their exclusion seems reasonable but does not validate the estimate that we used. If the catchment population were underestimated, this would give rise to a systematic overestimate of fracture probabilities both for hip fracture and major osteoporotic fracture, and vice versa. It is relevant, however, that, accuracy errors have little impact on the rank order with which the FRAX tool categorizes risk within a given population [12, 40] but they do change the absolute number generated and thus have implications where treatment guidelines are based on cost-effectiveness or the economic burden of disease.

In summary, a FRAX model has been created for the Republic of Uzbekistan that based on a regional population-based estimate of the incidence of hip fracture. The model should enhance accuracy of determining fracture probability among the Uzbek population and help to guide decisions about treatment.
